# Free-Radical Copolymerization of Dibenzofulvene with (Meth)acrylates Leading to π-Stacked Copolymers

**DOI:** 10.3390/polym10060654

**Published:** 2018-06-11

**Authors:** Jiyue Luo, Yue Wang, Tamaki Nakano

**Affiliations:** 1 Institute for Catalysis (ICAT) and Graduate School of Chemical Sciences and Engineering, Hokkaido University, N 21, W 10, Kita-ku, Sapporo 001-0021, Japan; luo@polymer.cat.hokudai.ac.jp (J.L.); yue.wang@polymer.cat.hokudai.ac.jp (Y.W.); 2 Integrated Research Consortium on Chemical Sciences (IRCCS), Institute for Catalysis (ICAT), Hokkaido University, N21 W10, Kita-ku, Sapporo 001-0021, Japan

**Keywords:** π-stacked polymer, dibenzofulvene, conformation, methacrylate, acrylate, radical polymerization, fluorescence, excimer, cyclic voltammetry

## Abstract

Copolymerizations of dibenzofulvene (DBF) with methyl methacrylate (MMA), 2-hydroxyethyl methacrylate (HEMA), methyl acrylate (MA), and 2-hydroxyethyl acrylate (HEA) were conducted under free radical conditions in toluene using α,α′-azobisisobutylonitrile (AIBN) as the initiator. In the copolymerizations, DBF indicated much higher reactivity than the comonomers, and the products comprised mainly of DBF units. NMR, UV, and fluorescence spectra, as well as electrochemical features indicated that the copolymers possess both isolated and rather short, sequential (meth)acrylate units, as well as π-stacked and unstacked DBF sequences. Isolated (meth)acrylate units are proposed to be sandwiched between DBF units. The ratios of π-stacked and unstacked side-chain fluorene groups of DBF units in excited states were accurately determined on the basis of fluorescent emission spectra; DBF units are mostly π-stacked in excited states as disclosed by fluorescence spectra. Two types of π-stacked sequences were suggested to be present in the ground state by electrochemical analysis. The copolymers exhibited higher solubility than pure poly(DBF).

## 1. Introduction

**Scheme 1:**
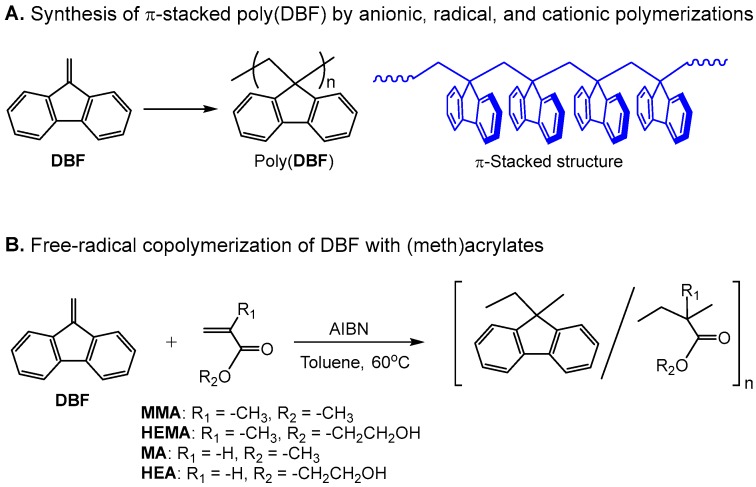
Synthesis of π-stacked poly(DBF) by anionic, radical, and cationic polymerization (**A**) and free-radical copolymerization of DBF with MMA, HEMA, MA, and HEA (**B**).

Polymer chain conformation plays important roles in macromolecular materials. A helix is one typical example of controlled chain conformation, and wide varieties of helical polymers have been synthesized and characterized [1,2,3,4,5]. Preferred-handed helical polymers find applications on the basis of their chirality for the fields of separation, catalysis, and photo-electronic materials. On the other hand, as another type of controlled conformation, a π-stacked structure was introduced; poly(dibenzofulvene) (poly(DBF)) was the first example of vinyl polymer having a π-stacked structure [6,7,8]. Poly(DBF) can be prepared by anionic, radical, and cationic polymerization of DBF [7,8,9,10,11,12,13] (Scheme 1A). The examples of π-stacked polymers include poly(DBF) and derivatives [6,7,8,9,10,11,12,13,14,15], poly(benzofulvene) and derivatives [16,17], polymers consisting of cyclophane-based monomeric units [18,19,20], polyurethanes [21], polyphenanthrolines [22], polyethers [23], and side-chain aromatic vinyl polymers [24,25]. Poly(DBF) indicates intriguing photo-electronic properties including characteristic photo-absorbance and emission profiles and rather high charge mobility due to the π-stacked side-chain fluorene moieties, and its derivatives with substituents on the fluorene backbone of the DBF unit have been synthesized. However, copolymerization of DBF with other types of monomers have not been reported so far in spite of the fact that copolymerization is a simpler method than the new monomer designs to modify polymer properties. Further, poly(DBF) tends to be insoluble in solvents when the degree of polymerization (DP) becomes high while oligomers are soluble. Copolymerization of DBF with other monomers may be expected to improve solubility by incorporation of flexible units.

In this work, knowing these backgrounds, we copolymerized DBF with conventional, (meth)acrylic monomers, i.e., methyl methacrylate (MMA), methyl acrylate (MA), 2-hydroxyethyl methacrylate (HEMA), and 2-hydroxyethyl acrylate (HEA) aiming to obtain copolymers in which π-stacked poly(DBF) sequences and comonomer sequences co-exist (Scheme 1B). The ratios of π-stacked and unstacked side-chain fluorene groups were accurately determined on the basis of fluorescent emission spectra, while such determination was difficult using ^1^H NMR spectra. In addition, electrochemical analyses lead to more detailed information on the π-stacked structure of the copolymers.

## 2. Materials and Methods

### 2.1. Materials

DBF was prepared according to the literature [8]. α,α’-Azobisisobutylonitrile (AIBN) (Wako Chemical, Osaka, Japan) was recrystallized from EtOH. Toluene (Wako Chemical, Osaka, Japan) was purified by washing with H_2_SO_4_ and distilled from Na wire in the presence of benzophenone. MMA (Wako Chemical, Osaka, Japan), MA (Wako Chemical, Osaka, Japan), HEMA (TCI, Tokyo, Japan), and HEA (TCI, Tokyo, Japan) were washed with aq. KOH, dried on MgSO_4_, and distilled under N_2_. Hexane (Kanto Chemical, Tokyo, Japan), tetrahydrofuran (THF) (Kanto Chemical, Tokyo, Japan), chloroform (Kanto Chemical, Tokyo, Japan), and diethyl ether (Kanto Chemical, Tokyo, Japan) were used as purchased.

### 2.2. Instrumentation

NMR spectra were recorded on JEOL JMN-ECX400 (400 MHz for ^1^H). UV–VIS spectra were taken using quartz cells on JASCO V-550 and V-570 (Tokyo, Japan) spectrophotometers. IR spectra were recorded with a JASCO FT/IR-6100 (Tokyo, Japan) spectrometer. Thermal analysis was conducted using RIGAKU Thermo Plus DSC8230 and TG8120 (Tokyo, Japan) apparatuses. Circular dichroism (CD) spectra were taken with a JASCO-820 (Tokyo, Japan) spectrometer. Emission spectra were taken on a JASCO FP-8500 (Tokyo, Japan) fluorescence spectrophotometer. Size-exclusion chromatography (SEC) measurements were carried out using a chromatographic system consisting of a JASCO DG-980-50 (Tokyo, Japan) degasser, a HITACHI L-7100 (Tokyo, Japan) pump, a HITACHI L-7420 (Tokyo, Japan) UV–VIS detector, and a HITACHI L-7490 (Tokyo, Japan) RI detector, equipped with TOSOH TSKgel G3000H HR and G6000H HR columns (30 × 0.72 (i.d.) cm) connected in series (eluent: THF, flow rate: 1.0 mL/min). Preparative recycling SEC was performed with a JAI LC-9201 (Tokyo, Japan) chromatograph consisting of a JAI PI-50 (Tokyo, Japan) pump and a Soma S-3740 (Tokyo, Japan) UV–VIS detector equipped with JAIGEL 1H and 2H columns (60 × 2 (i.d.) cm) connected in series (eluent: CHCl_3_, rate: 3.5 mL/min). Cyclic voltammetry was performed using an ALS/CH Instruments 630C (Tokyo, Japan) electrochemical analyzer using Pt-made working and counter electrodes with an Ag/AgCl standard electrode.

### 2.3. Determination of Monomeric Unit Ratios of Copolymers

**Figure A1:**
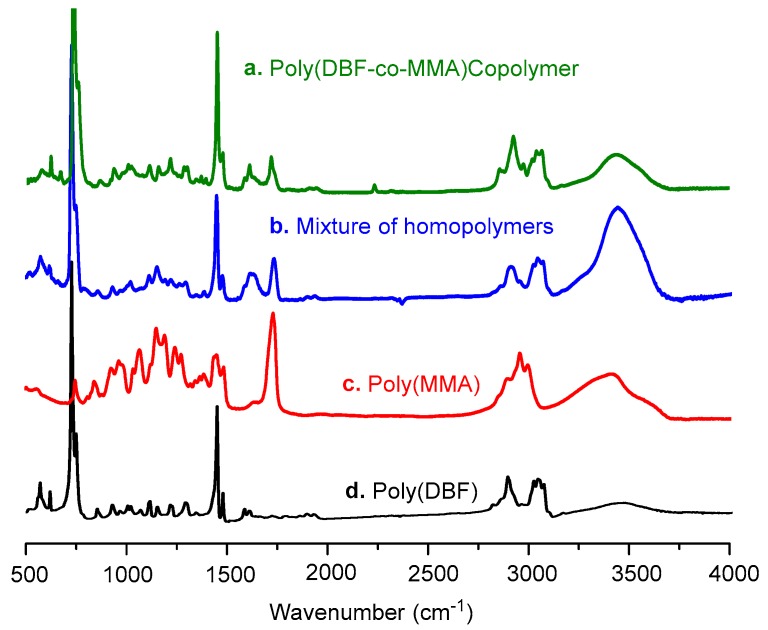
IR spectra of poly(DBF-*co*-MMA) (run 2 in Table 1, [DBF]/[MMA] = 82/18 in polymer) (a), a mixture of poly(DBF) (run 17 in Table 1) and poly(MMA) (run 1 in Table 1) at a residue molar ratio of 85/15 (b), poly(MMA) (run 1 in Table 1) (c), and poly(DBF) (run 17 in Table 1) (d). [KBr pellet].

**Figure A2:**
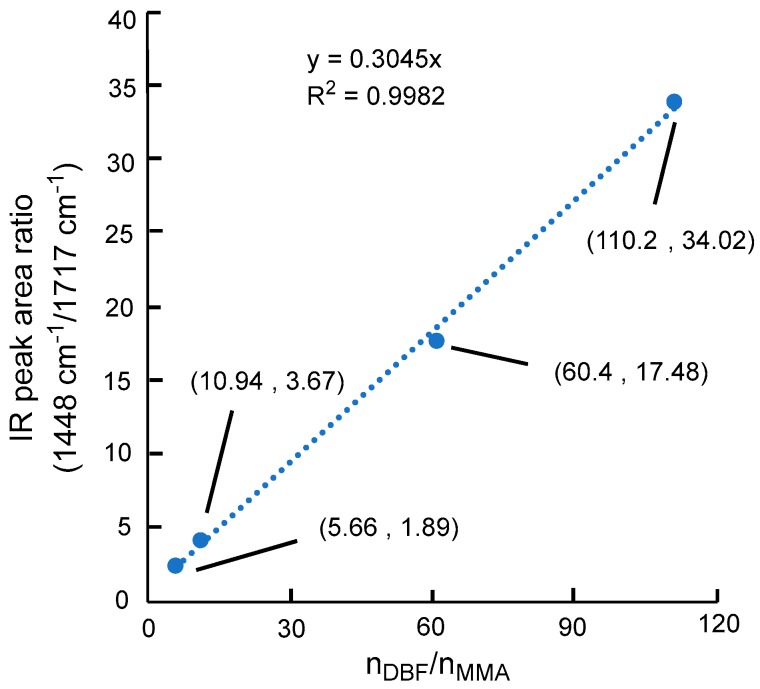
Linear function approximation of the data in Figure 1A through least squares regression.

Poly(MMA) (run 1 in Table 1) (19.3 mg, 0.193 mmol (per residue)) was dissolved in CHCl_3_ (3.85 mL). Four solutions of poly(DBF) (run 17 in Table 1) at different concentrations were prepared by dissolving the polymer of the following amounts, 9.8 (0.055 mmol), 10.8 (0.060 mmol), 9.7 (0.055 mmol), and 10.1 mg (0.057 mmol), in CHCl_3_ (1.50 mL), to which 0.01, 0.02, 0.1, and 0.2 mL, respectively, of the poly(MMA) solution was added. The mixed solutions were diluted by adding 1.5 mL of CHCl_3_. The resulting solutions were mixed with KBr powder, and the mixture was dried and fabricated to form a pellet. The pellet samples were subjected to IR analysis (Figure A1). Peak area ratios of IR signals at 1448 cm^−1^ (poly(DBF)) and at 1717 cm^−1^ poly(MMA) were plotted against molar ratio of monomeric residue ([DBF]/[MMA]); the plot was approximated with a linear equation, [peak area ratio] = 0.3045 × [unit ratio], through the least squares regression method where R^2^ was 0.998 (Figure A2).

For the other copolymers, single-point calibration was applied where poly(DBF) (10.6–10.9 mg) and relevant poly[(meth)acrylate] (5.11~18.9 mg) were dissolved in CHCl_3_ (3.00 mL), the solution was mixed with KBr powder, and the mixture was dried and fabricated to form a pellet.

### 2.4. Synthesis

#### Radical Copolymerization

A typical procedure is described for the copolymerization of DBF with MMA at [DBF]/[MMA] = 20/80 (run 2 in Table 1). DBF (712.0 mg, 4.00 mmol), MMA (1605.0 mg, 16.05 mmol), and AIBN (164.0 mg, 1.00 mmol) were dissolved in toluene (18.40 mL) in a glass ampoule equipped with a three-way stop cock under N_2_. After the solution was heated at 60 °C for 24 h in the dark, the reaction was quenched on cooling at 0 °C. The reaction mixture was poured into 400 mL of THF, and THF-insoluble part was collected with a centrifuge (5.7 mg, 0.2%). THF-soluble part was further fractionated by reprecipitation in hexane (400 mL), and the hexane-insoluble part was collected with a centrifuge (7.4 mg, 0.3%). The hexane-soluble part was recovered by removing solvents (800.5 mg, 34.5%) and was subjected to further purification by preparative SEC.

In the copolymerizations using MMA and MA, the THF-soluble part was fractionated into hexane-insoluble and -soluble parts while, in the reactions using HEMA and HEA, the THF-soluble part was fractionated into diethyl ether-insoluble and -soluble parts.

### 2.5. Computational Method

Molecular mechanics structure optimization was effected using the COMPASS [26] force field implemented in the Discover module of the Material Studio 4.2 (Accelrys, San Diego, CA, USA) software package with the Fletcher-Reeves [27] conjugate gradient algorithm until the RMS residue went below 0.01 kcal/mol/Å. Molecular dynamics simulation was performed under a constant NVT condition in which the numbers of atoms, volume, and thermodynamic temperature were held constant. Berendsen’s thermocouple [28] was used for coupling to a thermal bath. The step time was 1 fs and the decay constant was 0.1 ps. Conformations obtained through MD simulations were saved in trajectory files every 5 or 10 ps and were optimized by MM simulation.

## 3. Results

### 3.1. Copolymerization Reaction

The conditions and results of polymerization are summarized in Table 1. The reaction systems became heterogeneous in the course of polymerization due to the precipitation of insoluble products. The products were fractionated into the three parts, namely: (i) the THF-insoluble part; (ii) the THF-soluble, hexane-insoluble part (poly(DBF-*co*-MMA), and poly(DBF-*co*-MA)) or THF-soluble, diethyl ether-insoluble part (poly(DBF-*co*-HEMA) and poly(DBF-*co*-HEA)); and (iii) the hexane-soluble part (poly(DBF-*co*-MMA) and poly(DBF-*co*-MA)) or diethyl ether-soluble part (poly(DBF-*co*-HEMA) and poly(DBF-*co*-HEA)). The THF-soluble, hexane- or diethyl ether-insoluble part has higher molar mass than the hexane-soluble parts.

**Figure 1:**
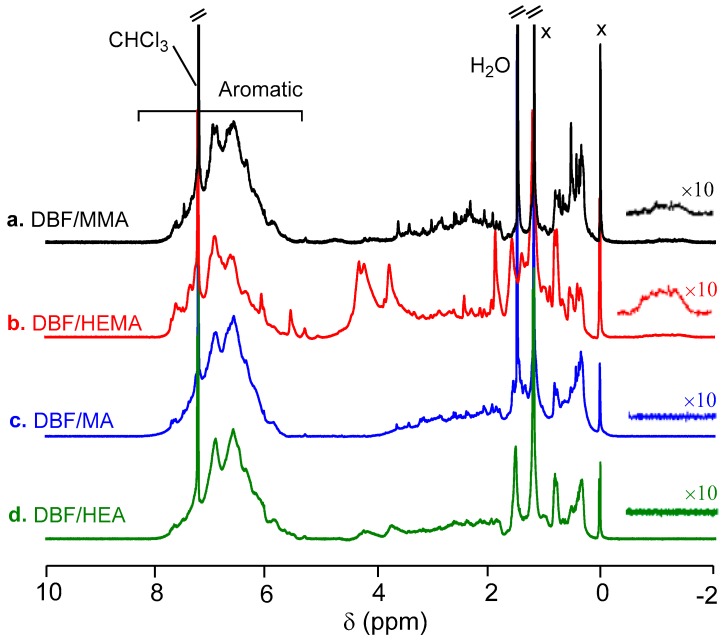
^1^H NMR spectra of poly(DBF-*co*-MMA) ([DBF]/[MMA] in polymer = 82/18) (a), poly(DBF-*co*-HEMA) ([DBF]/[HEMA] in polymer = 73/27) (b), poly(DBF-*co*-MA) ([DBF]/[MA] in polymer = 87/13) (c), and poly(DBF-*co*-HEA) ([DBF]/[HEA] in polymer = 89/11) (d) (400 MHz, CDCl_3_, room temperature). X denotes impurity.

The ratios of comonomer units in the copolymer were determined by IR spectra using homopolymers, i.e., poly[(meth)acrylate]s (runs 1, 5, 9, 13 in Table 1) and poly(DBF) (run 17 in Table 1), as standard samples (Figure A1 in Appendix A) whereas ^1^H NMR spectra indicated partially-overlapped signals which were not suitable for accurate determination of the ratios (Figure 1). As the yields of THF-soluble, hexane-insoluble part and THF-soluble, diethyl ether-insoluble part were lower than those of the hexane-soluble part and diethyl ether-soluble part, the latter products were mainly subjected to analyses. Additionally, the yields of the latter copolymer products were generally higher than that of soluble poly(DBF) (run 17 in Table 1); solubility is thus improved by copolymerization though the yield of the hexane- or diethyl ether-soluble parts was as high as 26%, and the *M*_n_s of this part were less than 1000.

While the homopolymerizations of (meth)acrylate monomers (runs 1, 5, 9, 13 in Table 1) and that of DBF (run 17 in Table 1) led to rather high monomer conversion, conversions of both DBF and (meth)acrylates in copolymerizations were lower than in the homopolymerizations, indicating that DBF and comonomers indeed form copolymers, not mixtures of homopolymers and that growing species with M_1_-M_2_ or M_2_-M_1_ units at the chain terminal has lower reactivity than that in homopolymerization systems due possibly to steric reasons.

In addition, conversions of MA in the copolymerizations were slightly, but clearly, higher than those of the three other comonomers in the corresponding copolymerizations. This may arise from the least bulky structure of MA among the four comonomers.

In the copolymerizations with all comonomers, the major products were insoluble in THF at [DBF]/[comonomer] = 80/20; the THF-insoluble products appeared to be almost pure homopolymers as their IR spectra matched that of poly(DBF) prepared by homopolymerization. At [DBF]/[comonomer] = 50/50 and 20/80, contributions of THF-soluble parts were more significant than at [DBF]/[comonomer] = 80/20. As indicated in run 17 in Table 1, homopolymerization of DBF leads to mostly THF-insoluble products. These results indicate that the copolymerization of DBF with acrylic monomers is indeed an effective way to synthesize polymers having DBF sequences with higher solubility than homopolymer of DBF.

### 3.2. Structrure of Copolymers

In all copolymerizations, the hexane-soluble part and diethyl ether-soluble part were rich in DBF units; even the copolymers prepared at [DBF]/[comonomer] (in feed) = 20/80 had the ratio of [DBF] units in the range of 73 to 89. These results mean that DBF is much more reactive than the acrylic comonomers in the copolymerizations.

Structures and properties of the hexane-soluble part and diethyl ether-soluble part are discussed hereafter. Figure 1 shows the ^1^H NMR spectra of poly(DBF-*co*-MMA), poly(DBF-*co*-MA), poly(DBF-*co*-HEMA), and poly(DBF-*co*-HEA) obtained at [DBF]/[comonomer] in feed = 20/80. The spectral shapes are different from those of poly(DBF) and poly[(meth)acrylate] (homopolymers), confirming that the copolymerization products are not mixtures of homopolymers. The spectra exhibit aromatic proton signals in the range of 5.5–7.8 ppm while fluorene, as a monomeric unit model, indicates aromatic signals at 7.3, 7.4, 7.5, and 7.8 ppm. The aromatic signals of the polymers in the range of 5.5–7.3 ppm are, thus, significantly up-field shifted, indicating that part of fluorene moieties of DBF units are stacked on top of each other (π-stacked conformation). The ratio of π-stacked DBF units to unstacked DBF units can be roughly estimated to be ca. 9/1 for the copolymers while more accurate estimation was difficult due to signal overlapping and broadening. In addition, the spectra did not show clear signals of rather long sequences of (meth)acrylate units which would be expected in the range of 1–2.5 ppm where main-chain methylene and methine signals, as well as α-methyl signals of homopolymers of (meth)acrylates should be observed. Instead of such signals, rather complicated signals appeared in the range of 0–1 ppm which may arise in part from (meth)acrylate units in the copolymers and in part from terminal groups originating from AIBN fragments. These observations may mean that sequences of (meth)acrylate units are very short comprising of only up to a few units surrounded by DBF units as the major components of the copolymer chain.

Further, it is noteworthy that poly(DBF-*co*-MMA) and poly(DBF-*co*-HEMA) indicated a rather broad signal centered at around −1 ppm. Since poly(DBF-*co*-MA) and poly(DBF-*co*-HEA) did not show such a signal, it may be based on α-methyl group. α-Methyl group of isolated, single methacrylate sandwiched by DBF units may be located on a benzene ring of the DBF unit which can exert significant magnetic anisotropic effects leading to large up-field shifts. On the basis of the monomeric unit ratios determined by IR spectra and the NMR signal intensities, the ratio of such isolated methacrylate units is estimated to be 13% for poly(DBF-*co*-MMA) and 23% for poly(DBF-*co*-HEMA) among methacrylate units in the copolymers.

### 3.3. Photophysical Properties of Copolymers

**Figure 2:**
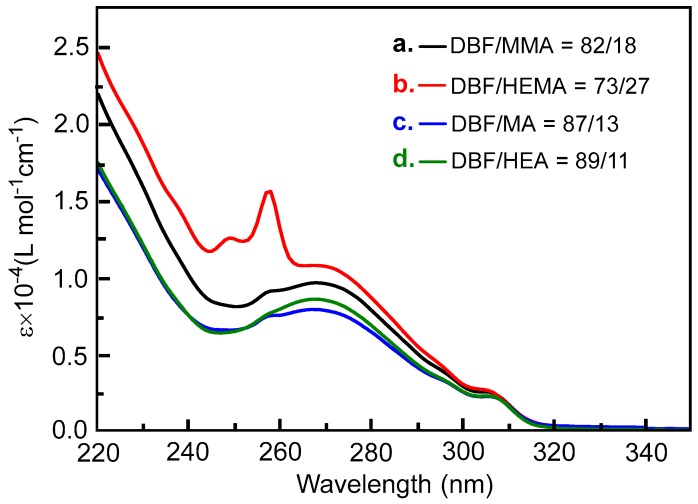
UV absorbance spectra of poly(DBF-*co*-MMA) ([DBF]/[MMA] in polymer = 82/18) (a), poly(DBF-*co*-HEMA) ([DBF]/[HEMA] in polymer = 73/27) (b), poly(DBF-*co*-MA) ([DBF]/[MA] in polymer = 87/13) (c), and poly(DBF-*co*-HEA) ([DBF]/[HEA] in polymer = 89/11) (d) ([DBF residue] = 0.94~1.15 × 10^−5^ M, THF, 1-cm cell, room temp.).

Absorbance spectra of copolymers prepared at [DBF]/[comonomer] in feed = 20/80 are shown in Figure 2. The absorbance spectral shapes of the copolymers are similar to those of poly(DBF)s prepared by radical polymerization under various conditions [11]. Further, molar absorptivities of the copolymers calculated with respect to DBF monomeric residue were much smaller than that of fluorene as a monomeric unit model. We have already established that the π-stacked structure leads to hypochromism for poly(DBF) and its derivatives and, also, it is known that π-stacked base pairs in the DNA double helix result in hypochromism [29,30,31]. The UV spectra thus support that the copolymers have π-stacked DBF sequences, as concluded through the NMR analyses; however, accurate extents of stacking are difficult to be determined by the UV spectra as well as by the NMR spectra since absorbance ranges of π-stacked and unstacked (isolated) DBF units overlap.

**Figure 3:**
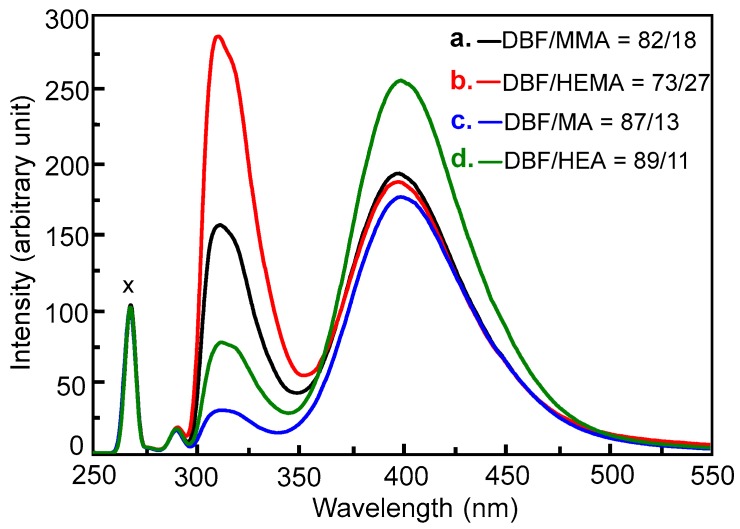
Fluorescent emission spectra of poly(DBF-*co*-MMA) ([DBF]/[MMA] in polymer = 82/18) (a), poly(DBF-*co*-HEMA) ([DBF]/[HEMA] in polymer = 73/27) (b), poly(DBF-*co*-MA) ([DBF]/[MA] in polymer = 87/13) (c), and poly(DBF-*co*-HEA) ([DBF]/[HEA] in polymer = 89/11) (d) [λ_ex_ = 267 nm, [DBF residue] = 0.94~1.15 × 10^−5^ M, THF, 1-cm cell, room temp.]. X denotes the excitation light signal.

Figure 3 shows the fluorescent emission spectra of the copolymers prepared at [DBF]/[comonomer] (in feed) = 20/80. In the fluorescent emission spectra of the copolymers, the emission peak position is considered to reflect whether the side-chain fluorene moiety is isolated or dimerized. The fluorescent emission spectra of the copolymers indicated sharper bands in the range of 300–330 nm which matches that of monomer emission of fluorene in addition to broad bands centered at around 400 nm whose shape and position are very similar to the excimer (dimer) emission bands of fluorene [32]. The former bands are, therefore, assigned to isolated fluorene units in the copolymer chain and the latter bands to π-stacked sequences. Since the two bands are well resolved, the ratios of π-stacked and unstacked (isolated) fluorene moieties in the copolymers can be unambiguously determined using the fluorescence spectra. As for the two bands, we have reported emission quantum yields, i.e., 0.69 for monomer emission and 0.06 for π-stacked, dimer emission of poly(DBF) prepared by radical polymerization [11]. On the basis of these quantum yields and the peak area ratios of the two types of emission bands, the unit ratios of π-stacked and unstacked (isolated) fluorene units were unambiguously determined to be 97/3 for poly(DBF-*co*-MMA) ([DBF]/[MMA] in polymer = 82/18), 90/10 for poly(DBF-*co*-HEMA) ([DBF]/[HEMA] in polymer = 73/27), 99/1 for poly(DBF-*co*-MA) ([DBF]/[MA] in polymer = 87/13), and 99/1 for poly(DBF-*co*-HEA) ([DBF]/[HEA] in polymer = 89/11). There seems to be a tendency that a higher DBF unit ratio in polymer leads to a higher ratio of π-stacked fluorene units regardless of the type of comonomer. Further, poly(DBF-*co*-MA) and poly(DBF-*co*-HEA) had 99% of π-stacked conformation while they have 13% and 11% of comonomer units, respectively. In these two copolymers, π-stacked DBF sequences and rather short acrylate sequences compose the chain with a very small amount of unstacked DBF units. However, these results should be carefully interpreted because possible energy transfer from isolated DBF unit to stacked DBF unit might underestimate the isolated unit ratio.

### 3.4. Electrochemical Properties of Copolymers

**Figure 4:**
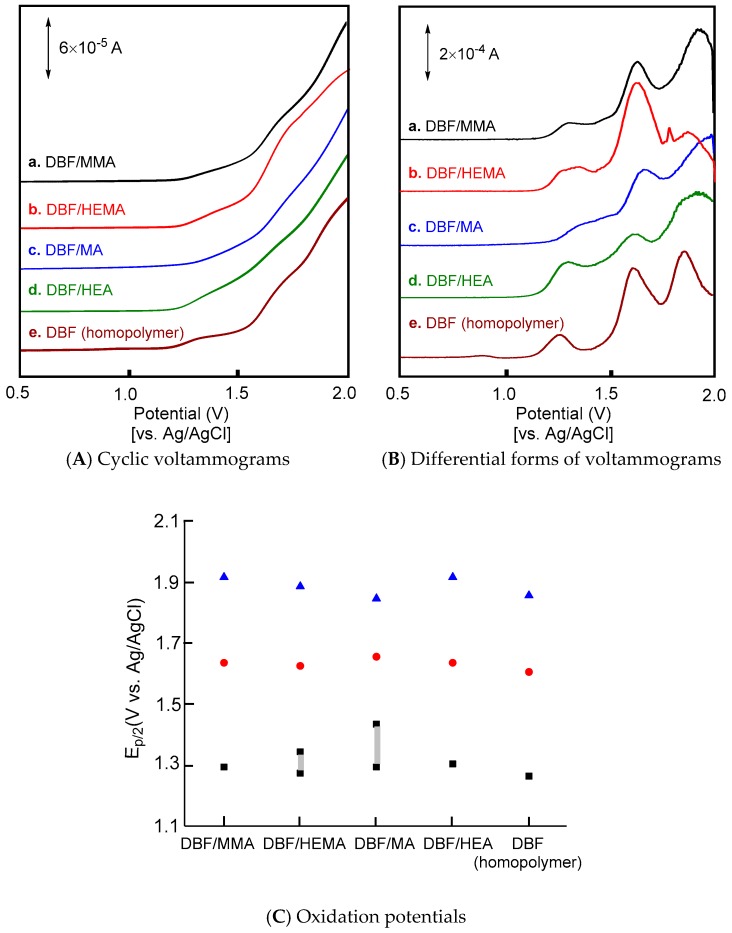
Cyclic voltammetry (CV) profiles (**A**), their differential forms (**B**) and oxidation potentials summary (**C**) of poly(DBF-*co*-MMA) ([DBF]/[MMA] in polymer = 82/18) (a), poly(DBF-*co*-HEMA) ([DBF]/[HEMA] in polymer = 73/27) (b), poly(DBF-*co*-MA) ([DBF]/[MA] in polymer = 87/13) (c), and poly(DBF-*co*-HEA) ([DBF]/[HEA] in polymer = 89/11) (d) (CV measurement conditions: CH_2_Cl_2_ solution containing *n-*Bu_4_NPF_6_, room temp.).

Figure 4A shows cyclic voltammetry (CV) profiles of copolymers obtained at [DBF]/[comonomer] (in feed) = 20/80. The profiles indicated three inflection points on the oxidation scan which are considered to correspond to oxidation potentials. In order to clearly identity the oxidation potentials, differential functions derived from the CV curves were used for analysis (Figure 4B).

Three oxidation potentials were found for all copolymers, and they are graphically summarized in Figure 4C. The highest oxidation potentials (ca. 1.8–1.9 V) may be ascribed to unstacked/isolated DBF units since fluorene, as a model of monomeric units, has been reported to have a higher oxidation potential (ca. 1.7 V vs. Ag/AgCl) than π-stacked DBF (fluorene) units. The observed potentials which are higher than that of fluorene may arise from interactions between the unstacked DBF unit and neighboring (meth)acrylate units where the aromatic group and carbonyl group may be in contact. Such interactions have been reported for a polyether having alternating aromatic-carbonyl junctions [23].

The two other, lower oxidation potentials are ascribed to π-stacked sequences. The positions of the second highest oxidation potentials (ca. 1.6 V vs. Ag/AgCl) are less than that of monomeric fluorene as a model of unstacked units (ca. 1.7 V vs. Ag/AgCl) by ca. 0.1 V, are even lower than that of the π-stacked dimer, and are comparable to that of the π-stacked trimer reported for isolated oligomers prepared by anionic polymerization [8]; the oxidation occurring at around 1.6 V may be based on the rather fragmented, shorter π-stacked structure. On the other hand, the lowest oxidation potentials (1.3–1.4 V vs. Ag/AgCl) are even lower than that of longer π-stacked sequences (ca. 1.5 V vs. Ag/AgCl) prepared by stereochemically-regulated anionic polymerization [8]; the oxidation occurring at this potential may be ascribed to the rather longer π-stacked structure. The shorter and longer π-stacked sequences appear to be electrochemically independent.

The three oxidation potentials were also observed for poly(DBF) prepared by radical polymerization. The occurrence of the shorter and longer π-stacked sequence may arise from the stereochemical nature of radical polymerization which is generally much less controlled than anionic polymerization.

While the second highest oxidation potential due to the shorter π-stacked DBF sequence seems to be almost unaffected by the chemical structure, the highest potential (unstacked DBF units) and the lowest potential (longer π-stacked DBF sequence) appear to vary depending on the polymer structure (Figure 4C). Poly(DBF-*co*-HEMA) and poly(DBF-*co*-MA) had broader ranges of the lowest oxidation potential than the other copolymers, suggesting that longer π-stacked DBF sequences have less homogeneous conformational features in the copolymers. In addition, it may be pointed out that poly(DBF-*co*-MA) has clearly higher values for the lowest oxidation potential compared with the other copolymers. It could be interpreted that the least bulky comonomer, MA, which is considered to be most reactive among the four comonomers, tends to make π-stacked DBF sequences in the copolymer chain shorter than the other comonomers.

Although the copolymers were indicated to have mostly π-stacked DBF sequences by the fluorescent spectra and the homopolymer of DBF also has been reported to possess mostly π-stacked conformation, their π-stacked conformations were found to be less homogeneous from an electrochemical view than that of poly(DBF) prepared by anionic polymerization, which shows only one oxidation signal in cyclic voltammetry [8]. Electrochemical analysis was, thus, found to be an effective method to shed light on conformational characteristics of aromatic polymers where different oxidation potentials can be ascribed to distinctive conformations.

### 3.5. Thermal Properties of Copolymers

**Figure 5:**
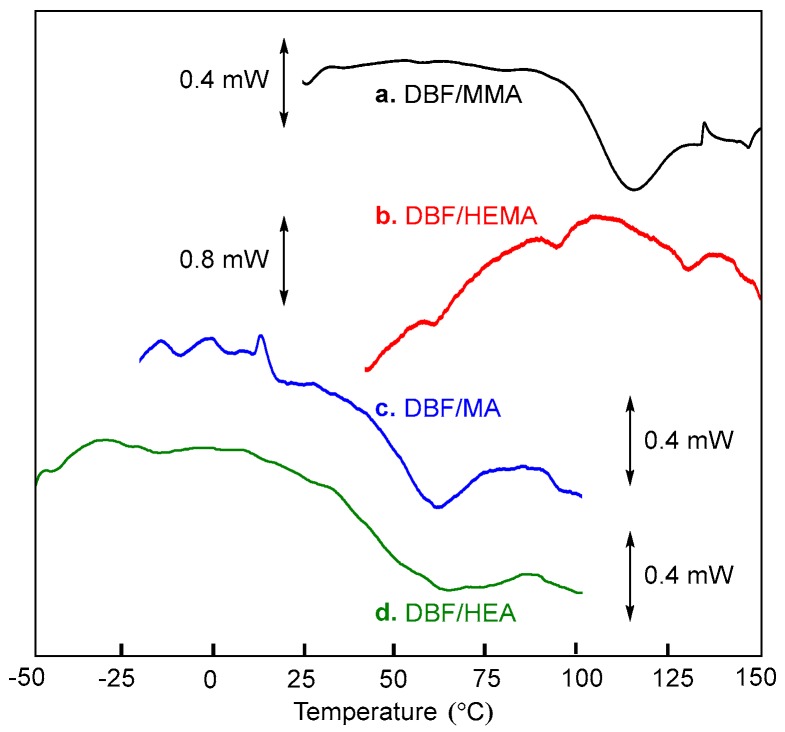
DSC profiles of poly(DBF-*co*-MMA) ([DBF]/[MMA] in polymer = 82/18) (6.7 mg) (a), poly(DBF-*co*-HEMA) ([DBF]/[HEMA] in polymer = 73/27) (4.6 mg) (b), poly(DBF-*co*-MA) ([DBF]/[MA] in polymer = 87/13) (4.3 mg) (c), and poly(DBF-*co*-HEA) ([DBF]/[HEA] in polymer = 89/11) (4.3 mg) (d) (second heating scan at a rate of 10 °C/min). Intensity of all profiles has been normalized to a sample amount of 10.0 mg.

Differential scanning calorimetry (DSC) profiles of the copolymers are shown in Figure 5. poly(DBF-*co*-MMA), poly(DBF-*co*-MA), and poly(DBF-*co*-HEA) exhibited clear glass transition temperatures (Tgs) at 100, 37, and 26 °C, respectively. The copolymers’ Tgs are higher than the Tgs of the homopolymers of corresponding comonomers, i.e., 88 °C for poly(MMA), 7 °C for poly(MA), and −30 °C for poly(HEA). These results suggest that the copolymers may have more rigid chains than the homopolymers of comonomers; rigidity of π-stacked DBF sequences of the copolymers seems to significantly contribute to the copolymers’ thermal properties.

Poly(DBF-*co*-HMEA), on the other hand, did not indicate a clear Tg and showed three minor endothermic peaks, while poly(HEMA) shows Tg at 116 °C. This may mean that the presence of π-stacked DBF sequence significantly affects the polymer’s thermal properties. Aggregation between π-stacked DBF sequences, in addition to inter-chain hydrogen bonding, might be responsible for such a result.

### 3.6. Proposed Structure of Poly(DBF-co-MMA)

**Figure 6:**
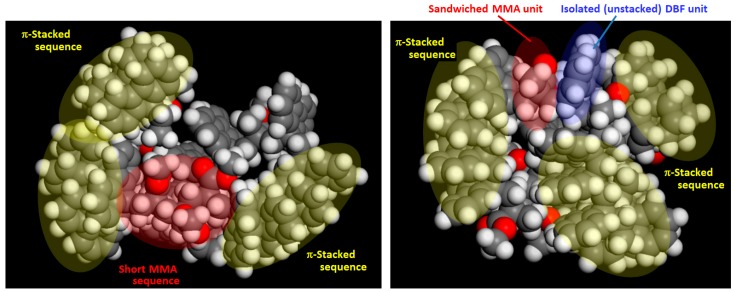
Poly(DBF-*co*-MMA) structure obtained through MD simulations for ca. 20 ns at 300 K: views from two angles.

A molecular model for poly(DBF-*co*-MMA) was created considering the following characteristics suggested through the experiments: (1) a chain comprising mostly of π-stacked DBF sequences which are fragmented by short MMA sequences; (2) the presence of isolated DBF units; and (3) the presence of isolated MMA units. The model consisting of 17 DBF units and six MMA units were conformationally equilibrated through molecular dynamics (MD) simulations for 20 ns at 300 K (Figure 6). The simulated model comprises of π-stacked DBF sequences, an isolated (unstacked) DBF unit, a short MMA sequence (three units in the figure), and a sandwiched MMA unit, which is consistent with the interpretation of the experimental results discussed so far. The model suggests that the presence of MMA units does not deteriorate π-stacking of DBF units in the vicinity. Additionally, the α-methyl group of the MMA units next to the DBF unit may be located on the top of the benzene ring where magnetic anisotropy results in up-field shifts; this character is more significant for the sandwiched MMA unit. The isolated (unstacked) DBF unit does not strongly interact with the other π-stacked DBF units, but may have interactions with the carbonyl group of neighboring MMA units. The simulated structure appears to be much more random and flexible in conformation than that of poly(DBF) [8], while the copolymer still possesses π-stacked DBF sequence conformation basically intact.

## 4. Conclusions

Copolymers of DBF with MMA, MA, HEMA, and HEA were prepared by free radical random copolymerization. The copolymers were significantly rich in DBF units regardless of the ratio of DBF to comonomer in the feed. The obtained copolymers showed higher solubility compared with poly(DBF). The copolymers comprised mainly of π-stacked sequences of DBF units in addition to a minor amount of unstacked DBF (fluorene) units as well as rather short comonomer units sequences and isolated, single comonomer units sandwiched by DBF units. The ratios of π-stacked and unstacked DBF sequences were estimated on the basis of the fluorescence spectra; most DBF units were revealed to be π-stacked. Electrochemical analyses suggested that the copolymers comprised of three different structures of DBF units, isolated ones, and π-stacked ones with longer and shorter sequences. Such structural heterogeneity may arise from less effective stereochemical control of radical polymerization compared with anionic polymerization. The discrepancies between the results from fluorescent and electrochemical analyses may be related to differences in structure between the ground state and excited states. The copolymers and poly(DBF) prepared by radical polymerization have a less controlled π-stacked structure compared with that of poly(DBF) prepared by anionic polymerization in the ground state, while the conformation of the radical polymerization products seem to become more homogeneous in excited states than in the ground state. Such a change may possibly be triggered by excimer formation on photo-excitation.

We may conclude that copolymerization of DBF with methacrylates and acrylates can lead to π-stacked polymer materials which have higher solubility due to structural flexibility of an entire chain introduced by the rather short (meth)acrylate sequences which connect π-stacked DBF sequences with different electrochemical characters. This aspect may lead to characteristic photo-electronic properties that may not be achieved by poly(DBF) prepared by anionic polymerization. Further, if functional methacrylates and acrylates are used as comonomers, functionalities may be able to be introduced to π-stacked polymer materials to widen the scope of application.

## Figures and Tables

**Table 1:** Radical copolymerization of DBF (M_1_) and methacrylates and acrylates (M_2_) in toluene at 60 °C for 24 h using AIBN ^a^.

Run	M_2_	[M_1_]/[M_2_] in Feed	Conv. (%) ^b^		Hexane-Soluble or Diethyl Ether-Soluble Part ^c,d^				Hexane or Diethyl Ether-Insoluble, THF-Soluble Part			THF-Insoluble Part
			M_1_	M_2_	Yield ^e^ (%)	*M*_n_^f^	*M*_w_/*M*_n_^f^	[M_1_]/[M_2_] in Polymer ^g^	Yield (%)	*M*_n_^f^	*M*_w_/*M*_n_^f^	Yield (%)
1	MMA	0/100	0	97	2	640	1.10		82	3570	2.33	~0
2		20/80	42	8	21	730	1.18	82/18	~0	1120	1.11	~0
3		50/50	56	5	10	850	1.16	90/10	9	1160	1.17	19
4		80/20	72	5	8	790	1.22	82/18	11	1310	1.19	45
5	HEMA	0/100	0	>99	4	410	1.51		2	1540	1.29	90
6		20/80	56	11	26	670	1.52	73/27	2	1280	1.15	2
7		50/50	60	5	25	940	1.40	88/12	11	1490	1.14	23
8		79/21	71	4	2	830	1.26	93/7	11	1470	1.19	56
9	MA	0/100	0	97	8	400	1.18		86	14,880	2.16	~0
10		20/80	42	17	25	620	1.10	87/13	~0	1620	1.52	~0
11		50/50	54	13	18	790	1.13	96/4	8	1250	1.19	17
12		80/20	69	13	5	590	1.21	94/6	10	1300	1.23	50
13	HEA	0/100	0	>99	5	360	1.05		87	1430	1.13	~0
14		20/80	48	5	18	700	1.19	89/11	1	2750	1.43	~0
15		50/50	60	4	12	950	1.19	95/5	5	1670	1.13	17
16		80/20	74	3	3	870	1.22	96/4	14	1820	1.12	57
17	None (DBF homo-polymerization)	100/0	78	0	9	790	1.14		2	1490	1.13	70

^a^ DBF weight = 0 mg (run 1), 712 mg (run 2), 854 mg (run 3), 712 mg (run 4), 0 mg (run 5), 187 mg (run 6), 445 mg (run 7), 712 mg (run 8), 0 mg (run 9), 178 mg (run 10), 445 mg (run 11), 748 mg (run 12), 0 mg (run 13), 178 mg (run 14), 445 mg (run 15), 676 mg (run 16), 890 mg (run 17); [DBF] = 0 M (run 1), 0.2 M (run 2), 0.48 M (run 3), 0.80 M (run 4), 0 M (run 5), 0.21 M (run 6), 0.50 M (run 7), 0.80 M (run 8), 0 M (run 9), 0.2 M (run 10), 0.50 M (run 11), 0.84 M (run 12), 0 M (run 13), 0.2 M (run 14), 0.5 M (run 15), 0.76 M (run 16), 1. 0 M (run 17). ^b^ Determined by ^1^H NMR analysis of the reaction mixture. ^c^ For runs 1–4, 9–12, and 17, the solvent of reprecipitation was hexane. For runs 5–8 and 13–16, the solvent of reprecipitation was diethyl ether. ^d^ This fraction was purified by preparative SEC once (eluent CHCl_3_) except for the products of homo-polymerizations. ^e^ Calculated excluding the contribution of unreacted monomer found by ^1^H NMR analysis. ^f^ Determined by SEC (eluent THF) using standard polystyrene samples. ^g^ Determined by IR spectra analysis.
